# Expert Consensus on Tiered Diagnosis and Treatment of Breast Cancer as a Single‐Disease Management Model in China (2025 Edition)

**DOI:** 10.1002/cai2.70031

**Published:** 2025-10-23

**Authors:** Jiani Wang, Shuping Wang, Erdan Huang, Fei Ma

**Affiliations:** ^1^ Department of Medical Oncology National Cancer Center/National Clinical Research Center for Cancer/Cancer Hospital, Chinese Academy of Medical Sciences and Peking Union Medical College Beijing China; ^2^ National Health Development Research Center Beijing China

**Keywords:** breast cancer, expert consensus, functional role, patient‐centered care, tiered diagnosis and treatment

## Abstract

Breast cancer is the most prevalent female malignancy worldwide. In 2022, China recorded approximately 357,000 new breast cancer cases and 75,000 deaths, posing a serious threat to female health. Given the “inverted pyramid” structure of China's healthcare system in service provision and resource distribution, there is an urgent need to establish a rational medical service framework that can optimize resource allocation, ensure patients receive appropriate diagnosis and treatment at different stages, and strengthen patient‐centered continuity of care, thereby enhancing overall therapeutic outcomes. Under the tiered diagnosis and treatment framework, the Breast Cancer Expert Committee of the National Cancer Quality Control Center and the National Health Development Research Center of the National Health Commission have jointly drafted and formulated this *Expert Consensus on Tiered Diagnosis and Treatment of Breast Cancer as a Single‐disease Management Model of Breast Cancer in China (2025 Edition)* to clarify the functional roles of different tiers of healthcare institutions, rationally allocate medical resources, and establish an integrated healthcare service system. This system emphasizes primary care, two‐way referrals, differentiated management of acute and chronic conditions, and coordinated multi‐level collaboration. The goal is to optimize comprehensive breast cancer management, spanning prevention, screening, diagnosis, treatment, and rehabilitation, ultimately improving patient survival and quality of life.

## Graphical abstract

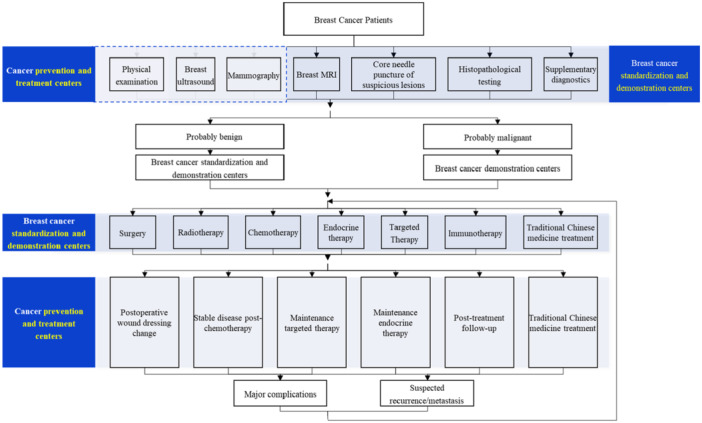

**Abbreviations**
MDT: multidisciplinary Team
MRI: magnetic Resonance Imaging

## 1 Overview

Breast cancer is the most prevalent cancer among women worldwide, with its incidence and mortality increasing annually in China, making it a critical public health issue that threatens female health [1]. While numerous clinical guidelines and consensus documents have been established worldwide for early screening, diagnosis, treatment, and standardized management of breast cancer [2, 3], leading to significantly improved patient prognosis and making breast cancer a model for chronic disease management in oncology, China faces unique challenges. The vast geographical disparities, uneven economic development, and unequal distribution of medical resources have compromised the continuity of care for some patients, resulting in delayed or suboptimal treatment.

In October 2024, the National Health Commission issued the *Notice on Strengthening Primary Care and Referral Services to Enhance the Continuity of Medical Services*, aiming to advance the tiered diagnosis and treatment system, further strengthen primary care and referral services, enhance the continuity of healthcare services, improve patients' healthcare experiences, and reduce healthcare costs. The document outlines phased objectives: By the end of 2025, a well‐functioning two‐way referral system will be established within compact medical consortia (including urban medical groups and county‐level medical communities). Additionally, an interinstitutional referral system will be established at the prefecture level to facilitate patient transfers within municipal jurisdictions. By 2027, a provincial‐level interinstitutional referral system will be established to facilitate seamless patient transfers within the province. By 2030, the tiered diagnosis and treatment system should operate effectively nationwide, delivering systematic, continuous, equitable, and accessible healthcare services and enabling a standardized healthcare‐seeking framework [4].

Under the guidance of the National Health Development Research Center and the Breast Cancer Expert Committee of the National Cancer Quality Control Center, the Chinese multidisciplinary breast cancer expert panel has formulated this consensus document based on the latest global medical evidence, therapeutic strategies, and clinical experience on breast cancer, addressing three key points: construction of a standardized tiered diagnosis and treatment system, comprehensive management from prevention, screening, diagnosis, treatment, to rehabilitation, and quality control metrics for tiered diagnosis and treatment. This consensus aims to clarify the responsibilities of healthcare institutions and professionals across different healthcare tiers, optimize resource allocation, promote equitable access to essential healthcare services, and guide patients in selecting appropriate facilities based on disease progression, enabling breast cancer patients to receive high‐quality, safe, appropriate, and continuous services throughout the course of breast cancer treatment.

## 2 Current State of Tiered Diagnosis and Treatment in China

### 2.1 Epidemiological Profile of Breast Cancer in China

In 2022, China reported 357,200 new breast cancer cases and 74,900 deaths, making it the second most common malignancy in women and the fifth leading cause of cancer‐related mortality [5]. With continuous innovation in therapeutic agents and the establishment of molecular subtype‐based precision diagnosis and treatment models, the 5‐year survival rate for breast cancer patients in China has increased to 83.2%, with early‐stage cases exceeding 90% [6].

However, the 5‐year survival rate of breast cancer differs significantly across regions. Data from the National Cancer Center (2016) revealed a 77.8% 5‐year survival rate in urban areas versus 55.9% in rural regions, a difference exceeding 20% [7]. Rural patients also had higher recurrence rates compared to urban patients (41.3% vs. 34.8%) [8].

### 2.2 Current State of Breast Cancer Diagnosis and Treatment in China

Breast cancer prevention and treatment techniques have become increasingly sophisticated. The advancements in multidisciplinary approaches, including screening technology, surgery, radiotherapy, chemotherapy, targeted therapy, endocrine therapy, and immunotherapy, have significantly improved the survival and prognosis of breast cancer patients. With the implementation of multimodal treatment approaches, breast cancer has become one of the solid tumors with a favorable prognosis, facilitating the development of standard‐of‐care protocols.

A nationwide survey on the current state of breast cancer diagnosis and treatment and physicians' opinions across 185 hospitals in 30 regions of China revealed progressive quality control improvements in pilot hospitals and notable performance in screening and diagnostic metrics. Additionally, the leading and demonstrative roles of pilot hospitals across regions have helped raise clinicians' awareness of breast cancer management, aligning their practices with international standards and expert consensus [9].

### 2.3 Current State of Tiered Diagnosis and Treatment of Breast Cancer in China

Given China's large population and substantial breast cancer burden, tiered healthcare systems can effectively prevent resource congestion while optimizing their utilization. Since 2012, China has established 13 national medical centers specializing in different fields, along with 125 national and 114 provincial medical centers. Additionally, over 1400 diagnosis and treatment technologies have been transferred to provinces in need of assistance, leading to a stable reduction in cross‐provincial and cross‐regional medical visits despite growing national healthcare demands. Subsequent efforts will address regional disparities in healthcare system development and resource allocation.

Efforts to implement primary care have focused on standardizing primary healthcare institutions, promoting grassroots initiatives to deliver high‐quality services, developing general practitioner‐centered talent pools, and expanding family physician contracting services. In 2023, over 30,000 township health centers and community health stations met service capacity standards, handling 52% of national outpatient and emergency visits, gradually approaching the WHO‐recommended 80% primary care utilization rate [10].

Significant efforts have been made to enhance two‐way referrals and coordination across different tiers of healthcare institutions. This includes the development of medical consortia, such as urban healthcare groups and county‐level medical communities, as well as the formation of specialty alliances focused on under‐resourced clinical disciplines and the diagnosis and treatment of major diseases. These initiatives aim to expand access to high‐quality medical resources and guide healthcare institutions at all levels in strengthening multidisciplinary and collaborative service delivery. By the end of 2023, more than 18,000 medical consortia had been built nationwide, facilitating 30.32 million two‐way referrals and creating a new landscape of vertical medical resource flow and patient referrals. Notably, 45.6% of county hospitals have achieved Level 3 competencies, and 87.7% achieved Level 2 competencies [11]. Furthermore, the healthcare service capacity of county hospitals has steadily increased over the years. A survey conducted in Shanghai revealed that the majority of patients (64.9%) were willing to be referred from community to higher‐level hospitals, but not the reverse (53.3%) [12].

For the differentiation between acute and chronic care, China has established regional, provincial, and national medical centers, as well as emergency centers specializing in chest pain, stroke, and trauma to improve the diagnosis and treatment of acute, critical, and complex diseases, while innovating service models through telemedicine, internet healthcare, and home‐based care. These initiatives effectively address diverse needs across emergency, critical, and chronic care domains, promoting rational healthcare‐seeking behavior [13].

International tiered healthcare systems primarily encompass two models: the incentive‐and‐constraint‐based approach (e.g., Japan's model) that guides patient flow through market mechanisms [14], and the mandatory regulatory frameworks imposed by government or market forces (e.g., the UK model) [15, 16]. At present, there is no research data on the tiered diagnosis and treatment of breast cancer in China, and it is urgently needed to supplement it to facilitate the rational allocation of medical resources and achieve equal medical services.

## 3 Establishment of a Patient‐Centered Standardized Tiered Diagnosis and Treatment System for Breast Cancer

### 3.1 Constructing a Tiered Diagnosis and Treatment Service Network for Breast Cancer Patients

Advancements in diagnostic technologies, local/systemic therapies, and surgical techniques have transformed breast cancer care from a disease‐centered model to an innovative, patient‐centered approach. When constructing a tiered diagnosis and treatment system, factors such as patient feedback, informed consent, and shared decision‐making power should also be considered. The tiered network primarily comprises demonstration centers, standardization centers, and prevention and treatment centers, and aims to address the continuity of care within the chronic disease management framework for breast cancer. It promotes primary care triage, facilitates bidirectional referrals and multi‐tier collaboration, strengthens the differentiation between acute and chronic care, enhances patients' quality of life, and establishes optimized healthcare policies through social resources to deliver cost‐effective healthcare services.

### 3.2 Defining the Tiered Diagnosis and Treatment Process for Breast Cancer

#### 3.2.1 Developing a Three‐Tier Center Model With Refined Diagnosis and Treatment Pathway

By strategically coordinating existing medical resources, a three‐tier disease‐specific alliance consisting of breast cancer demonstration centers, standardization centers, and prevention and treatment centers will be established to integrate patients across administrative regions into the management framework.

#### 3.2.2 Strengthening Categorization, Stratification, and Referral Processes

Compact county‐level healthcare communities, serving as the operational backbone of prevention and treatment centers, will play a pivotal role in advancing population health. Efforts will focus on strengthening the development of county‐level grassroots tumor prevention centers, optimizing medical resource allocation, enhancing primary diagnostic capabilities, and ensuring effective distribution of quality resources at the grassroots level to achieve the strategic objectives of Healthy China.

**Figure 1:**
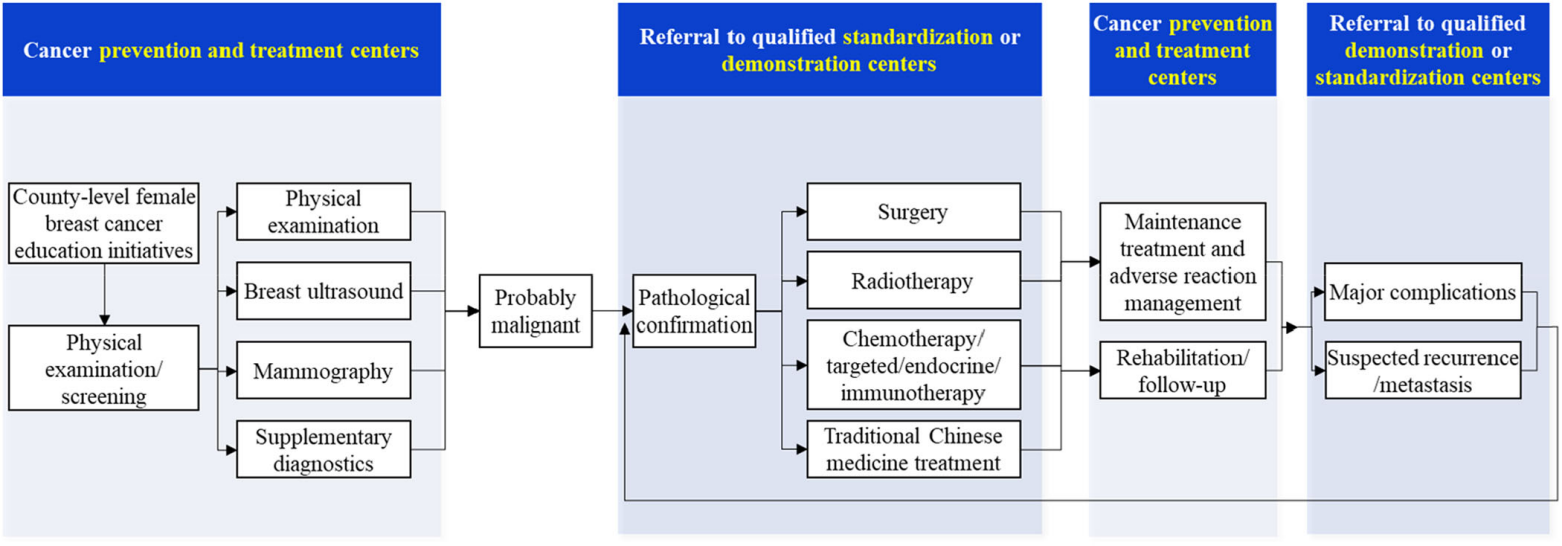
Tiered diagnosis and treatment pathway in cancer prevention and treatment centers.

Primary healthcare institutions, supported by county‐level prevention and treatment centers, will carry out screening services for key populations and promptly manage and refer patients according to the following tiered principles (Figure 1).

#### 3.2.3 Implementing Disease‐Specific Coordination and Case Management for Closed‐Loop Management

**Figure 2:**
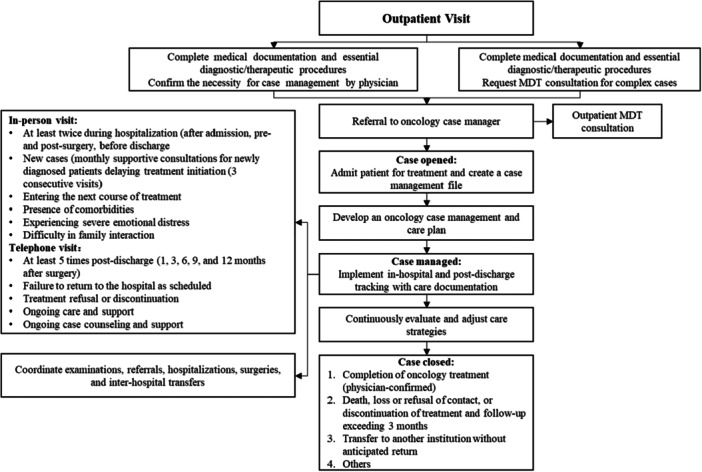
Breast cancer case management process and pathway.

To ensure patient‐centered, cross‐institutional collaboration throughout the process of tumor diagnosis, treatment, and management, disease‐specific coordinators will be designated across all tiers of medical centers to oversee patient referral coordination and medical record management (Figure 2).

Case managers are the cornerstone of integrated care in single‐tier medical institutions, overseeing the coordination of all processes from start to finish. Oncology case managers provide continuous medical services to patients, ensuring seamless transitions across treatment stages.

### 3.3 Defining the Functional Roles of Healthcare Institutions

**Figure 3:**
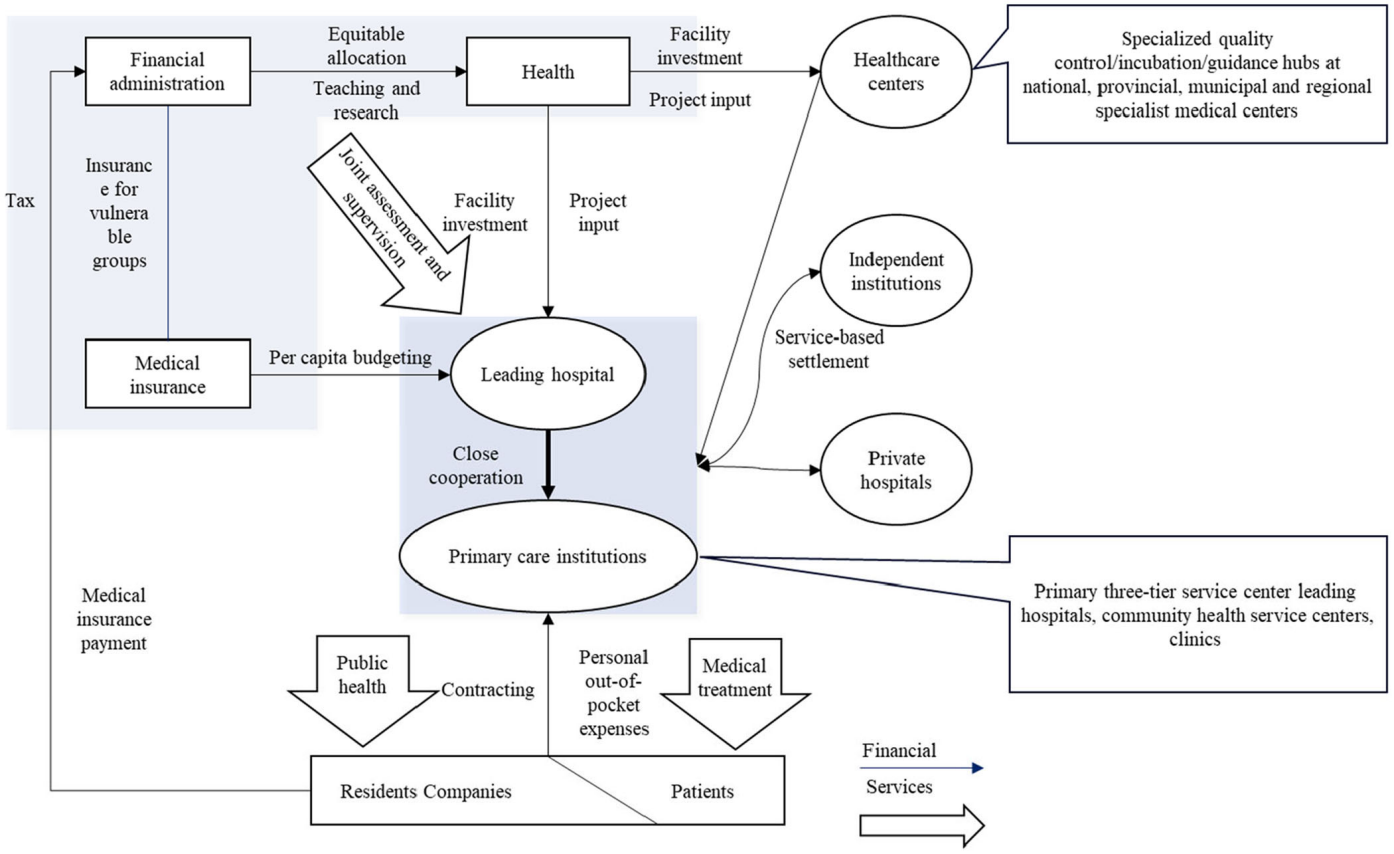
Structure of a two‐tier, six‐level integrated medical and health service system.

In response to China's aging population and the growing healthcare demands for chronic diseases, the nation will gradually implement a “two‐tier, six‐level” integrated service framework that aligns with the policy requirements for establishing a tiered diagnosis and treatment system and a compact medical consortium. The “two‐tier” structure consists of “Healthcare Centers” and “Health Management Platforms,” each subdivided into three levels. Healthcare Centers include national, provincial, and prefecture‐level specialized healthcare centers, focusing primarily on complex and severe diseases and undertaking quality control, clinical incubation initiatives, technological leadership, and innovation within specialized fields. Health Management Platforms encompass leading hospitals and primary healthcare institutions, and are divided into three tiers: county/district hospitals, township/community health service centers, and village clinics/community health service stations (Figure 3) [17].

The breast cancer center is divided into three levels: (1) Demonstration centers will manage complex and refractory cases, conduct clinical research and basic research transformation, provide technical guidance and professional training to standardization and prevention and treatment centers, as well as other healthcare institutions, and lead advancements in medical science, technological innovation, and talent development; (2) standardization centers at the prefecture‐city level will manage routine clinical diagnoses, develop personalized and standardized treatment plans per established guidelines and specifications, carry out annual specialized physical examinations and subsequent treatment of comorbidities, and oversee bidirectional referral processes; (3) prevention and treatment centers will provide rehabilitation and nursing services for patients with confirmed diagnoses, stable posttreatment conditions, and those in recovery phases. These centers will undergo regular quality and effectiveness assessments by higher‐tier institutions and, in accordance with diagnosis and treatment guidelines, collaborate with higher‐level hospitals for follow‐ups and disease monitoring, and implementation of treatment plans established by those hospitals.

### 3.4 Enhancing Standardized Diagnosis and Treatment of Breast Cancer

#### 3.4.1 Defining the Service Processes of Healthcare Institutions at All Levels

To strengthen and standardize the top‐down design, efforts should focus on two key areas: (1) Enhancing primary healthcare services by refining the institutional framework for initial visits at the primary level. This includes establishing case management systems, telemedicine platforms, and collaborative mechanisms among various eligible healthcare institutions to optimize bidirectional referrals and cooperation. (2) Standardizing medical consortia for specialized disease management by promoting telemedicine services, thereby enhancing the comprehensive service capabilities of county‐level hospitals.

1. Primary medical prevention and treatment centers provide services for diagnosed patients in stable or recovery phases.
  Service process: Receive patients and conduct preliminary diagnosis or assess referred patients → Develop a diagnosis and treatment plan within the institution's capacity → Determine whether the patient qualifies for tiered diagnosis and treatment services → If eligible, obtain informed consent from the patient → Establish a specialized disease archive → Conduct follow‐up visits, continuous treatment, physical examinations, and health management.
  Process for referral to higher‐level hospitals: A specialist determines that the patient meets the referral criteria → Fully communicates with the patient and/or family before referral → Determines the appropriate level of hospital referral based on the patient's condition → Contacts a secondary or higher‐level standardization/demonstration center → A specialist at the receiving hospital confirms the necessity of referral → Issues a referral slip or shares patient information with the receiving hospital through an information platform → Transfers the patient to a standardization/demonstration center.
2. Secondary or higher‐level standardization/demonstration centers provide medical services such as surgery, radiotherapy, and chemotherapy to breast cancer patients.

Process for newly diagnosed patients: Receive the patient and conduct diagnosis → Develop a treatment plan through Multidisciplinary Team (MDT) discussions → Provide active treatment (such as surgery, radiotherapy, and chemotherapy) → Assess whether the patient qualifies for tiered diagnosis and treatment services after disease stabilization → If eligible, transfer the patient to a primary healthcare institution for continued care → Regularly dispatch specialists to primary healthcare institutions for consultations and evaluations of tiered diagnosis and treatment quality.

**Figure 4:**
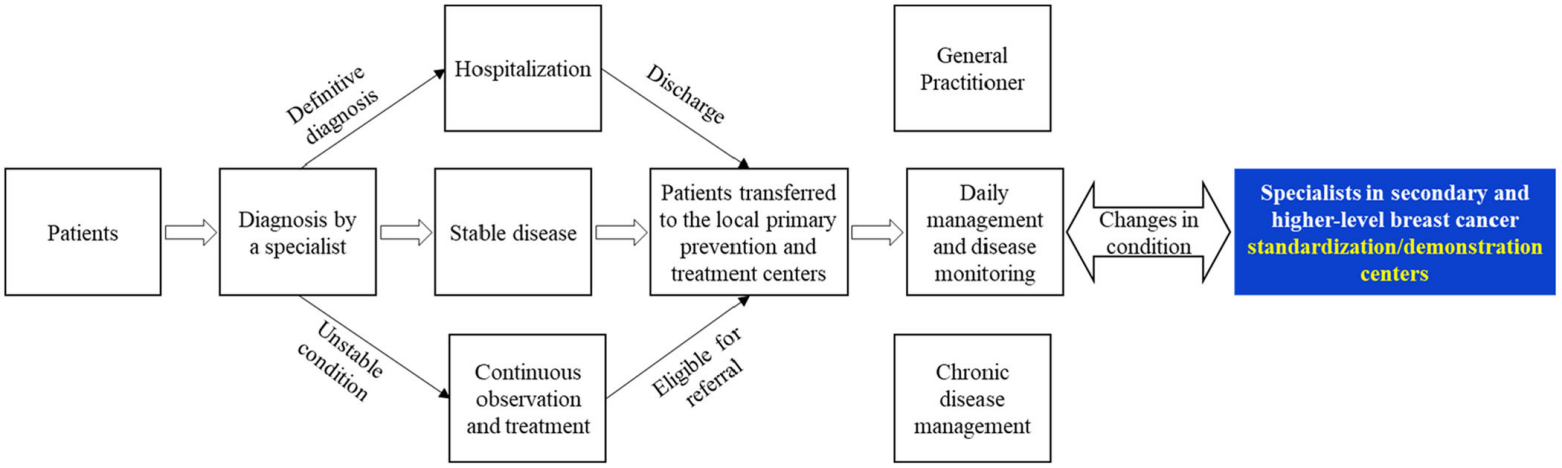
Service process in secondary and higher‐level standardization/demonstration centers.

**Figure 5:**
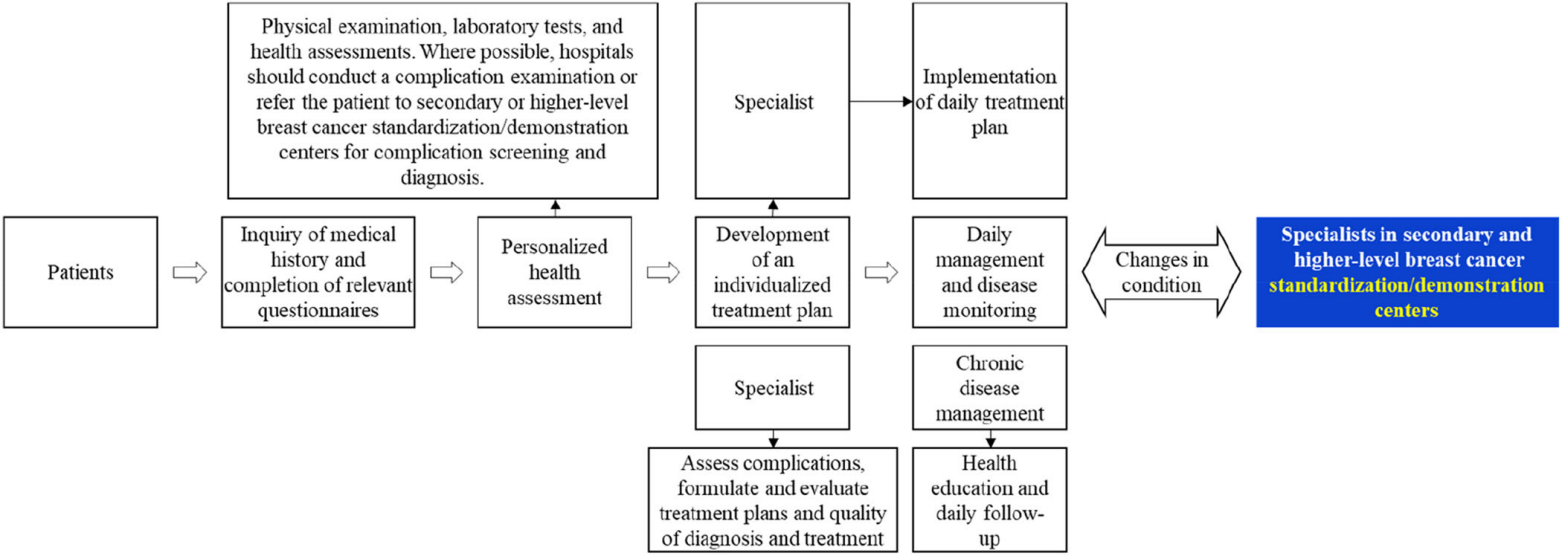
Individualized service process in secondary and higher‐level standard/demonstration centers.

Process for receiving upward and downward referrals: Receive the patient and conduct diagnosis → Develop a treatment plan through MDT discussions (including surgery, radiotherapy, and chemotherapy) → Determine if the patient has stable disease and meets the criteria for downward referral → Fully communicate with the patient and/or family before referral → Contact a primary healthcare institution → Submit a referral request or share patient information with the receiving institution via an information platform → Transfer the patient to a primary healthcare prevention and treatment center (Figures 4 and 5).

#### 3.4.2 Improving Healthcare Services by “Three‐Medicals Linkage”

A well‐integrated operational and management framework that coordinates medical care, insurance, and pharmaceuticals is crucial for improving the tiered diagnosis and treatment system for breast cancer. The goal is to strengthen the overall service capacity for breast cancer management. A composite payment model should be established, primarily based on single‐disease payment, supplemented by capitation payment and bundled payments per service unit. To ensure a seamless diagnosis and treatment process across different healthcare levels, it is necessary to eliminate disparities in medical insurance payment policies for breast cancer management. Outpatient and inpatient referrals that align with the tiered diagnosis and treatment system should be eligible for continuous deductible accumulation, facilitating the orderly movement of patients between different tiers of healthcare institutions.

Additionally, medical service pricing should be reasonably set and adjusted. Essential and innovative breast cancer drugs should be uniformly supplied and coordinated across all levels of healthcare institutions within the region. A fast‐track approval process for personalized medicines should be implemented to optimize drug management processes, ensuring the standardization of treatment plans for referred patients.

Higher‐level hospitals should provide referred patients with priority consultation, examination, and hospitalization. These hospitals are encouraged to formulate drug treatment plans and associated implementation procedures, which should then be implemented and managed by lower‐level hospitals or primary healthcare institutions. Through bidirectional referrals, a unified drug supply system, and a continuous composite insurance framework, a seamless referral process between different levels of healthcare institutions can be established.

#### 3.4.3 Strengthening Information Technology Construction to Achieve Smart Referral

A collaborative network spanning all levels of healthcare can be established through information technology infrastructure, creating an interconnected platform for cancer management. Ongoing refinement of the provincial‐level national oncology health information platform enhances seamless connectivity across all cancer center platforms. Electronic patient archives, electronic medical records, and basic resource databases should be developed to enhance data integration and business coordination across medical services, medical insurance, and drug supply systems.

To achieve smart referral, a strong emphasis will be placed on developing extensive data sets, which serve as the fundamental basis for information sharing and resource integration. By developing comprehensive, accurate, and timely medical health data sets, healthcare institutions will be supported in making informed decisions, while patients will benefit from personalized and continuous care. This will greatly enhance referral efficiency and precision, driving the development of a smart referral system.

##### 3.4.3.1 Data Set Components

Basic information (age, gender, date of birth, ethnicity, marital status, contact details, home address, Medical insurance type, and menstrual and pregnancy history), medical history [(past medical history (e.g., hypertension, coronary heart disease, diabetes, and thyroid disorders), surgical history, and allergy history], and family history (family history of breast cancer and other relevant diseases); diagnostic and treatment information: Screening results (breast ultrasound, mammography, and breast Magnetic Resonance Imaging [MRI]), diagnostic reports (clinical diagnosis, pathological diagnosis, and molecular phenotyping), treatment plans (surgical procedures, chemotherapy, radiotherapy, endocrine therapy, and targeted therapy), surgical records (surgery date, surgery name, surgical process, and intraoperative observations), and radiotherapy and chemotherapy data (chemotherapy cycles, chemotherapy dose, radiation dose, and target areas); rehabilitation information: Rehabilitation plans [(rehabilitation exercises and schedules and rehabilitation assessments (evaluation metrics and assessment timelines)], records of complications (type, time of occurrence, and treatment), and follow‐up information (dates, methods, and patient outcomes). Such data set thoroughly captures all aspects of breast cancer patient data, ensuring a systematic and complete record that spans from basic information to follow‐up care.

In addition, data collection should follow standardized procedures to ensure the accuracy and completeness of the data, and establish a dynamic update mechanism to enhance the scalability of the data, to adapt to the diversity of future research and diagnosis and treatment.

At present, China has successfully established the National Antitumor Drug Surveillance System, the country's largest oncology diagnosis and treatment database. This database covers 31 provinces and 1427 hospitals, with data dating back to 2013. It encompasses 870 types of drugs and complete health information on about 15 million patients, with 80% of them available for assessing overall survival outcomes. This comprehensive data set provides precise data support for China's cancer prevention and treatment efforts.

#### 3.4.4 Enhancing Major Chronic Disease Management Through a Three‐Tier Collaboration Network

Establishing a three‐tier oncology collaboration network (provincial, municipal, and county levels) leveraging information technology to integrate leading provincial institutions, regional diagnosis and treatment centers, and county‐level medical communities fosters cross‐institutional collaboration for multi‐center, multi‐tier, large‐cohort clinical studies, ensuring holistic management throughout the entire cycle of major chronic diseases, particularly breast cancer.

**Figure 6:**
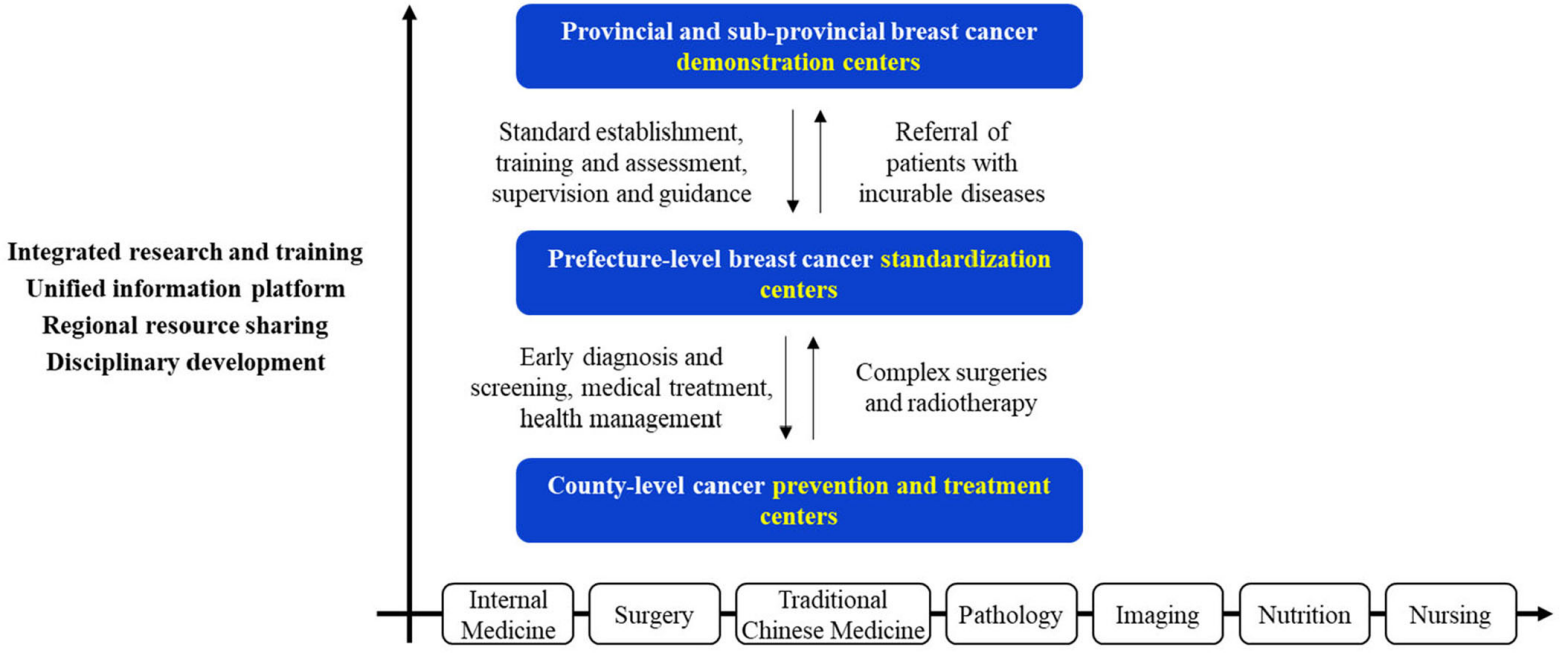
Three‐tier collaborative management pathway for breast cancer.

In line with the development strategy of the “Healthy China Initiative,” the three‐tier collaboration network prioritizes health life expectancy as its outcome, while focusing on standardized management rate (short‐term) and effective survival rate (long‐term) of major chronic diseases such as breast cancer as process milestones. Comprehensive assessments are carried out by analyzing key metrics, such as regional hospitalization rates, the proportion of primary‐level outpatient and emergency visits, the share of medical service revenue within the system, and the balance between medical insurance income and expenditure, all of which contribute to realizing the “Healthy China 2030” vision (Figure 6).

### 3.5 Patient‐Centered, Tiered Management Objectives, Pathways, and Bidirectional Referral Criteria for Breast Cancer

#### 3.5.1 Objectives

The objectives are to enhance breast cancer survival rates and improve patients' quality of life by leveraging the strengths of the three‐tier single‐disease breast cancer centers. This is achieved through efforts to raise awareness of breast cancer prevention and treatment, increase early diagnosis rates, standardize diagnostic and therapeutic approaches, strengthen the role of primary hospitals in prevention and care, ensure lifelong management, and collect patients' feedback for patients undergoing treatment.

#### 3.5.2 Pathways

**Figure 7:**
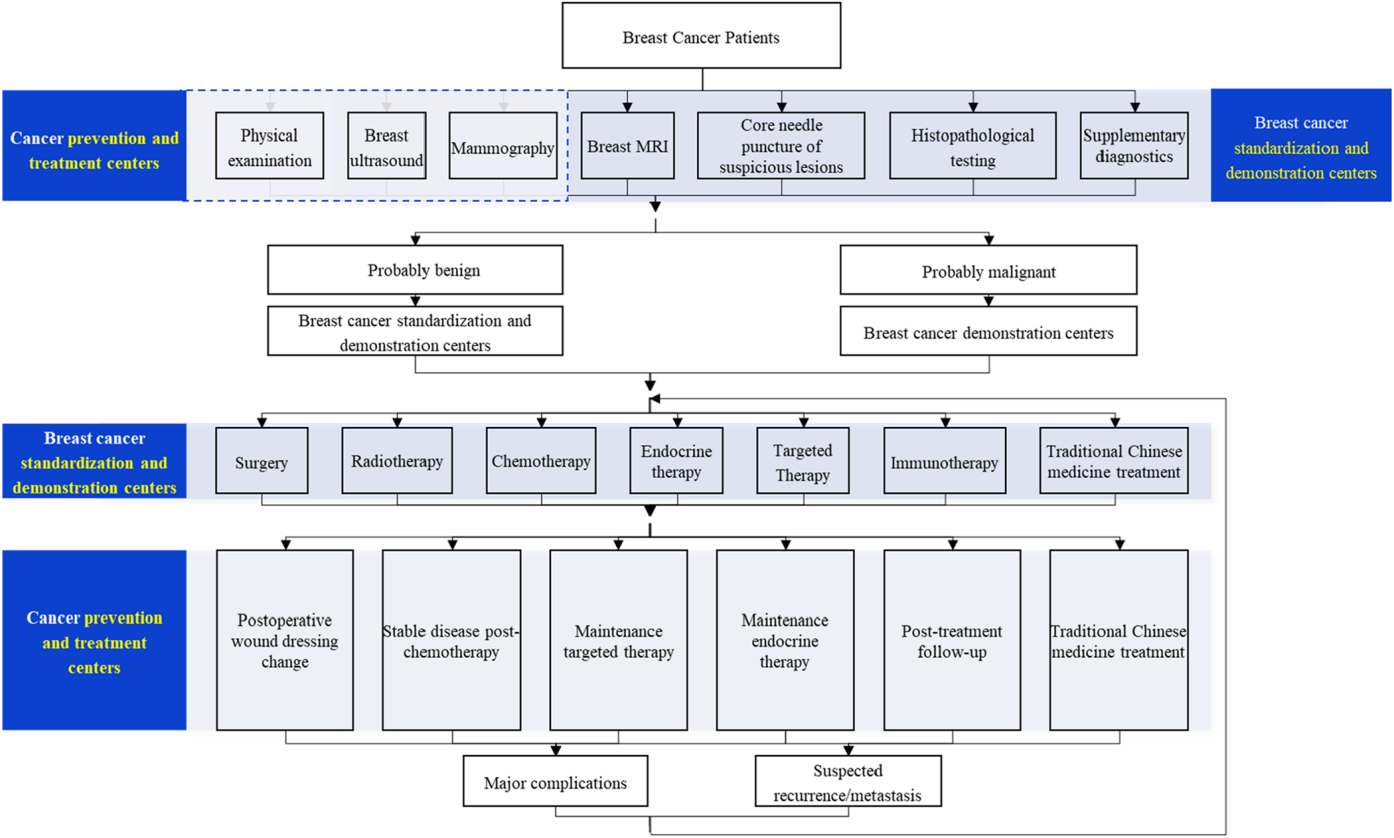
Tiered diagnosis and treatment pathway for breast cancer. *Note:* Other examinations include positron emission tomography‐computed tomography (PET‐CT) and laboratory tests (clinical chemistry and tumor marker detection).

 The tiered diagnosis and treatment pathway for breast cancer is designed with a patient‐centered approach. It is implemented through Cancer Prevention and Treatment Centers and Breast Cancer Standardization and Demonstration Centers, and classifies patients into appropriate clinical pathways based on their individual clinical profiles, ensuring a scientific and rational categorization process (Figure 7).

#### 3.5.3 Bidirectional Referral Criteria

##### 3.5.3.1 Criteria for Upward Referral to Standardization/Demonstration Hospitals

Patients newly diagnosed with breast disease at community‐level hospitals who present any of the following conditions: (1) Clinical and imaging examinations showing benign breast lesions that are indicated for surgery, are difficult to treat, or require differentiation from malignant diseases; (2) Clinical and imaging examinations suggesting breast disease with an undetermined benign or malignant nature; (3) Clinical and imaging examinations suggesting malignant breast disease; (4) Patients with undetermined pathology due to limited diagnostic capabilities in primary hospitals.

Patients with breast cancer undergoing follow‐up at community hospitals who present any of the following conditions: (1) Patients requiring postoperative dressing changes who experience changes in condition; (2) patients undergoing preoperative or postoperative chemotherapy who develop serious complications; (3) patients undergoing postoperative radiotherapy who develop serious complications; (4) patients receiving postoperative endocrine therapy who develop serious complications; (5) patients with suspected tumor recurrence or metastasis; (6) Patients with other conditions that cannot be managed adequately.

##### 3.5.3.2 Criteria for Referral to Breast Cancer Demonstration Hospitals

(1) Breast masses with clinical findings highly suspicious for breast cancer or an unclear diagnosis; (2) blood‐tinged nipple discharge; (3) ultrasound or mammography indicating BI‐RADS category IVB or higher or ultrasound revealing suspicious lymph nodes (such as localized cortical thickening and loss of lymph node hilum); (4) development of serious complications during breast cancer treatment; (5) suspected tumor recurrence or metastasis; (6) disease progression during treatment necessitating adjustment of treatment regimen; (7) no significant symptom relief after 2–4 weeks of multimodal treatment; (8) other conditions that cannot be managed adequately.

##### 3.5.3.3 Criteria for Downward Referral From Breast Cancer Demonstration Hospitals to Standardization Hospitals

(1) Postoperative wound dressing changes; (2) patients with wound or tumor ulceration requiring long‐term dressing changes; (3) rehabilitation following breast cancer treatment; (4) posttreatment follow‐up; (5) patients experiencing mild to moderate toxicities between chemotherapy sessions; (6) patients experiencing mild to moderate toxicities during endocrine or targeted therapy or immunotherapy; (7) chest wall or breast skin reactions following radiotherapy; (8) patients with mild conditions requiring inpatient observation.

##### 3.5.3.4 Criteria for Downward Referral to Primary Cancer Prevention and Treatment Centers

(1) Completion of treatment (surgery, radiotherapy, or chemotherapy), with stable disease and no signs of critical illness; (2) stable disease post‐multimodal treatment, requiring simple rehabilitation therapy.

#### 3.5.4 Specialist Referral Criteria

The diagnosis and treatment of breast cancer involve multidisciplinary collaboration among the surgery, internal medicine, radiology, pathology, imaging, ultrasound departments and mental health department. To optimize medical resource allocation, enhance diagnosis and treatment efficiency, and improve the quality of life of patients, a standardized tiered referral system for specialties must be established. Based on the *Guidelines for the management path and quality control of breast cancer prevention and treatment in China's counties (2023 Edition)* [18], the specific specialist referral criteria are as follows:

##### 3.5.4.1 Surgery

It is generally challenging for primary prevention and treatment centers to perform complex procedures such as breast‐conserving surgery and breast reconstruction. However, standardization or demonstration centers are typically equipped with more comprehensive technical expertise and advanced equipment. Referral decisions should be based on the complexity of the surgical procedure, hospital classification, technical proficiency, and the availability of appropriate equipment and facilities.

##### 3.5.4.2 Internal Medicine

Diagnostic methods at primary prevention and treatment centers are generally limited, with restricted access to novel therapeutic drugs. Referral decisions should be based on clinical staging, disease progression, and patient preference.

##### 3.5.4.3 Radiology

It may be challenging for primary prevention and treatment centers to perform advanced radiotherapy, with some centers lacking dedicated radiology departments. Referral decisions should be based on technical proficiency, availability of appropriate equipment and facilities, and patient preference.

##### 3.5.4.4 Pathology

Pathology departments in primary prevention and treatment centers are relatively underdeveloped. When necessary, tumor samples should be sent to pathology departments in breast cancer demonstration/standardization centers. Alternatively, remote pathology consultations can be conducted to ensure accurate histopathological and molecular profiling, which is essential for guiding treatment.

##### 3.5.4.5 Imaging

If imaging equipment or expertise in image interpretation is unavailable in primary prevention and treatment centers, or in the case of complex cases, patients should be referred to higher‐level facilities or undergo remote consultation.

##### 3.5.4.6 Ultrasound

If primary prevention and treatment centers lack adequate experience in ultrasound imaging and are unable to determine the benign or malignant nature of lesions, or encounter complex cases, patients should be referred to higher‐level facilities or managed via teleconsultation. Furthermore, if ultrasound‐guided biopsy cannot be performed in primary hospitals, patients should be transferred to a higher‐level hospital for further evaluation and management.

##### 3.5.4.7 Mental Health Department

If the primary prevention and treatment center lacks psychiatrists with sufficient experience and is unable to assess the patient's mental state and provide psychological intervention, the patient must be transferred or consulted remotely.

#### 3.5.5 Promoting the Application of Convenient Diagnostic and Treatment Models in Tiered Care

According to the *Chinese expert consensus on an innovative patient‐centered approach to diagnosis and treatment of cancer* [19], convenient diagnosis and treatment refer to various diagnostic and therapeutic activities provided by qualified healthcare institutions and personnel in multiple settings to optimize medical processes, streamline medical procedures, and ensure rapid access to medical and nursing services. As a key component of an innovative patient‐centered approach to diagnosis and treatment of cancer, this model includes several formats, such as daycare diagnosis and treatment centers (covering outpatient daycare clinics, daycare surgery, and daycare wards) and infusion centers (both inpatient and outpatient).

Daycare diagnosis and treatment are characterized by high efficiency, allowing for the optimized utilization of limited medical resources. This approach helps reduce the length of hospital stay, lower patient medical expenses, and improve the turnover rates of hospital beds, thus alleviating the challenge of bed shortages and achieving a mutually beneficial outcome for both patients and healthcare providers.

For certain patients requiring specialized medications, infusions or injections can be administered at qualified private clinics or dedicated community‐level infusion centers. This approach optimizes the administration processes, shortens treatment duration, and reduces financial burdens. In daycare infusion centers, patients receive professional hospital‐grade infusions with supported emergency services, requiring no inpatient admission. Once the infusion is complete and observation is concluded, patients can return home to rest.

Furthermore, advancements in drug formulations have facilitated the implementation of convenient diagnosis and treatment processes. Compared to prolonged intravenous infusions, the development and promotion of subcutaneous injection formulations allow patients to receive more convenient infusions within a shorter period of time. This reduces the risk of complications, decreases dependence on medical resources, and significantly enhances patient satisfaction with cancer treatment. Additionally, fixed‐dose drug administration, which eliminates the need for weight‐based (in kilograms) dosage calculations, plays a crucial role in reducing the risk of hospital‐acquired infections and improving medication safety. The simplified administration process also lowers the barriers for the use of targeted drugs, supports standardized treatment within the tiered diagnosis and treatment system, and enhances the quality of unified tiered diagnosis and treatment [20].

## 4 Diagnosis and Treatment Measures for Breast Cancer and Tiered Evaluation Metrics

### 4.1 Breast Cancer Prevention

Breast cancer prevention is classified into three levels. Primary prevention focuses on eliminating etiological or risk factors to prevent disease onset, representing the most proactive and fundamental approach. Secondary prevention aims at early detection, diagnosis, and treatment to identify the disease as soon as possible, to prevent or delay disease progression and worsening, and improve cure and survival rates. Tertiary prevention is rehabilitation‐oriented, providing multimodal treatments to enhance survival and recovery of diagnosed patients. This includes preventing tumor recurrence and metastasis, as well as managing postoperative pain and complications. Detailed prevention guidelines are outlined in relevant expert consensus [21].

### 4.2 Breast Cancer Screening

Breast cancer screening involves efficient, convenient, and cost‐effective breast examination methods to identify asymptomatic cases for early detection, diagnosis, and treatment, ultimately reducing breast cancer mortality in the general population. Screening methods include mammography, ultrasound, and MRI when necessary. Due to the lower penetrability of X‐rays in dense breast tissues and the breast characteristics of East Asian women, ultrasound is considered a routine screening method. MRI offers higher diagnostic accuracy but is more costly; thus, it is recommended as a supplementary examination to mammography and ultrasound. Specific screening procedures are detailed in relevant guidelines and expert consensus [22, 23].

### 4.3 Breast Cancer Diagnosis

This involves medical history collection, physical examination, laboratory tests, assessment of target organ damage, and molecular subtyping. However, a pathological diagnosis is required for breast cancer. Tissue samples can be obtained via needle or excisional biopsy, with needle biopsy being the preferred method in well‐equipped hospitals. Patients with suspected breast cancer should be informed of the biopsy results. Detailed diagnostic criteria are outlined in relevant clinical guidelines [24].

### 4.4 Breast Cancer Treatment

Breast cancer treatment encompasses surgery, radiotherapy, chemotherapy, targeted therapy, endocrine therapy, immunotherapy, and traditional Chinese medicine. Patients should be informed about standardized treatment options to instill confidence in achieving successful treatment, with the goal of managing breast cancer as a chronic condition. Specific treatment options are detailed in relevant guidelines [25, 26, 27, 28].

### 4.5 Posttreatment Rehabilitation Management of Breast Cancer

Rehabilitation management focuses on restoring physiological and psychosocial functions. Rehabilitation is provided during or after standard treatment, including all interventions to help patients regain physical function, adjust their mental state, and reintegrate into social life. Detailed rehabilitation management rules are found in relevant guidelines [29, 30].

### 4.6 Evaluation Metrics for Tiered Diagnosis and Treatment of Single Breast Cancer

To assess the implementation of clinical guidelines for standardized diagnosis and treatment of breast cancer and further improve quality control in breast cancer management, China has progressively developed a metric system for quality control of breast cancer. This consensus primarily refers to the following guidelines: *Quality control metrics for standardized diagnosis and treatment of breast cancer in China* [31], *Medical quality control metrics in cancer (2023 Edition)* [32], *National tertiary public hospital performance assessment manual (2024 Edition)* [33], *Annals of quality control for standardized diagnosis and treatment of breast cancer in China (2024 Edition)* [34], and Guidelines for the management path and quality control of breast cancer prevention and treatment in China's counties (2023 Edition) [18]. Different evaluation metrics are established for hospitals at various levels, as detailed in Tables 1, 2, 3, 4. Additional details on specific metrics are provided in Suppporting Information S1: Appendices S1–S3.

**Table 1:** Evaluation metrics for tiered diagnosis and treatment in breast cancer demonstration centers.

No.	Primary metrics	Secondary metrics
1	Management metrics	Rate of complications in patients undergoing breast cancer surgery
2		Rate of Class I surgical site infections in breast cancer surgeries
3		Rate of unplanned reoperation in breast cancer patients
4		Mortality rate among low‐risk breast cancer patients
5	Diagnostic metrics	Rate of clinical TNM staging assessment completion in patients with breast cancer before first treatment:
		Rate of clinical TNM staging in patients with breast cancer before first treatment
		Rate of standardization in clinical TNM staging in patients with breast cancer before first treatment
6		Rate of pathological diagnosis in patients with breast cancer before anticancer drug therapy
7		Rate of postoperative pathology report completeness in patients with breast cancer
8		Rate of pathological diagnosis in patients with breast cancer before radiotherapy
9		Rate of ultrasound correlation in patients with breast cancer before first treatment
10		Rate of mammographic correlation in patients with breast cancer before first treatment
11	Surgical metrics	Proportion of patients with early‐stage breast cancer undergoing sentinel lymph node biopsy
12		Proportion of patients with ≥ 10 axillary lymph nodes dissected
13	Radiotherapy	Proportion of patients with breast cancer receiving postoperative radiotherapy after breast‐conserving surgery
14		Proportion of patients with breast cancer receiving postoperative radiotherapy after modified radical mastectomy
15		Compliance rate of radiotherapy records for breast cancer patients
16	Pharmacological therapy	Proportion of patients with clinical stage III breast cancer receiving neoadjuvant therapy before surgery
17		Compliance rate of chemotherapy records for breast cancer patients
18		Proportion of patients with advanced metastatic (clinical stage M1) breast cancer receiving systemic treatment as initial treatment
19		Proportion of postoperative hormone receptor‐positive patients with breast cancer receiving adjuvant endocrine therapy
20		Proportion of HER2‐positive patients with breast cancer receiving postoperative targeted therapy
21		Proportion of hormone receptor‐positive patients with breast cancer receiving postoperative adjuvant endocrine therapy
22		Proportion of HER2‐positive patients with breast cancer receiving anti‐HER2 targeted therapy
23	Others	Proportion of patients with breast cancer undergoing MDT evaluation before first treatment
24		Follow‐up rate of patients with breast cancer after treatment

**Table 2:** Evaluation metrics for tiered diagnosis and treatment in breast cancer standard centers.

No.	Primary metrics	Secondary metrics
1	Management metrics	Rate of Class I surgical site infections in breast cancer surgeries
2	Diagnostic metrics	Rate of clinical TNM staging in patients with breast cancer before first treatment
3		Rate of standardization in clinical TNM staging in patients with breast cancer before first treatment
4		Rate of pathological diagnosis in patients with breast cancer before nonsurgical treatment
		Rate of pathological diagnosis in patients with breast cancer before anticancer drug therapy
		Rate of pathological diagnosis in patients with breast cancer before radiotherapy
5	Surgical metrics	Rate of sentinel lymph node biopsy in patients with early‐stage breast cancer
6		Rate of adequate axillary lymph node dissection
7	Radiotherapy	Rate of radiotherapy after breast‐conserving surgery in patients with breast cancer
8		Rate of radiotherapy after modified radical mastectomy in patients with breast cancer
9	Pharmacological therapy	Rate of neoadjuvant therapy in patients with locally advanced breast cancer
10		Rate of systemic treatment as initial treatment in patients with advanced metastatic breast cancer
11		Rate of postoperative adjuvant endocrine therapy in patients with hormone receptor‐positive breast cancer
12		HER2‐positive rate in patients with breast cancer receiving anti‐HER2 targeted therapy

**Table 3:** Evaluation metrics for tiered diagnosis and treatment in cancer prevention and treatment centers.

No.	Metrics
1	Rate of clinical staging before first treatment
2	Rate of postoperative pathological TNM staging
3	Rate of pathological diagnosis before first nonsurgical treatment
4	Rate of molecular pathology detection before first targeted therapy/immunotherapy
5	Compliance rate of intraoperative lymph node dissection

**Table 4:** Other reference requirements for tiered diagnosis and treatment cancer prevention and treatment centers.

No.	Category	Other reference requirements
1	Screening	Due to limited resources in primary medical institutions, breast ultrasound should be the preferred screening method in counties. If conditions permit, breast ultrasound and mammography are recommended. At the same time, remote imaging, mobile screening vehicles, and other methods can be considered to allocate human and material resources, supporting screening efforts at the county‐level and primary medical institutions.
2	Imaging diagnosis	Ultrasound, mammography, or MRI should be conducted for the breast and regional lymph nodes (axillary, supraclavicular, and infraclavicular regions).
3		Following a breast cancer diagnosis, additional imaging should be performed for other clinically indicated sites to evaluate the presence of distant metastases. Chest CT is recommended for patients diagnosed with breast cancer.
4		For patients with stage ≥ T3N1M0 or rapidly progressing disease, bone scintigraphy is recommended, which can also be used for routine screening and diagnosis of suspected bone metastases such as bone pain, fractures, alkaline phosphatase increased, or hypercalcemia.
5		If imaging equipment or expertise in image interpretation is unavailable at county‐level hospitals, or in the case of complex cases, patients should be referred to higher‐level facilities or undergo remote consultation.
6	Pathological diagnosis	The pathology report for breast cancer should clarify the benign or malignant nature of breast lesions and provide the following essential information: tumor size, histological type and grade, presence of ductal carcinoma in situ (DCIS), vascular nerve invasion, involvement of the nipple and surgical margins, lymph nodes, and expression of immunohistochemical markers such as estrogen receptor (ER), progesterone receptor (PR), HER2, and Ki‐67.
7		Considering the relatively slower development of pathology departments in county‐level hospitals, tumor samples may be sent to the pathology departments of higher‐level hospitals or certified third‐party laboratories when necessary. Remote pathology consultations can also be conducted to obtain accurate histopathological results and molecular profiling (such as HER2 expression or amplification) for guiding treatment.
8	MDT development	County‐level hospitals should actively organize and implement a multidisciplinary diagnosis and treatment approach for breast cancer, involving the (breast) surgery, medical oncology, radiation oncology, pathology, imaging, ultrasound departments and mental health department. It is recommended that multidisciplinary discussions be conducted before the initial treatment, either in the hospital, in collaboration with higher‐level hospitals, or through remote consultations, to ensure standardized and individualized anticancer treatment.
9	Surgical treatment	Before performing breast‐conserving surgery, hospitals should ensure that their pathology departments are capable of assessing surgical margins. If a patient is indicated for breast‐conserving surgery, which is challenging to perform at county‐level hospitals, referral to appropriately equipped hospitals at the same or higher level is recommended. Hospitals capable of performing pathological assessments and surgical procedures but lacking radiotherapy equipment and technology should refer patients to higher‐level hospitals for postoperative radiotherapy.
10		Due to the technical challenges of breast reconstruction and its limited application in county‐level hospitals, patients opting for total mastectomy with reconstruction needs should be referred to appropriately equipped hospitals at the same or higher level.
11		Sentinel lymph node biopsy (SLNB) requires appropriate tracers, as well as techniques and coordination among surgery, imaging, and pathology departments. Hospitals lacking this capability should refer patients eligible for SLNB to hospitals at the same or higher level with the necessary techniques and equipment.
12	Radiotherapy	Some county‐level hospitals may lack a dedicated radiation oncology department. It is recommended that referral pathways be established with the radiation oncology departments of hospitals at the same level or higher within the county to ensure timely access to postoperative adjuvant radiotherapy for patients.
13	Medical treatment	Some county‐level hospitals may not have access to drugs such as pertuzumab and neratinib. Thus, special medication procurement channels should be established for critical and major diseases with hospitals at the same or higher level within the county.
14		When treating advanced (stage IV) breast cancer, county‐level hospitals should comprehensively assess the patient's overall condition and confirm the current pathological diagnosis, molecular subtype, and TNM stage to develop a comprehensive anticancer treatment plan. Treatment plans should be developed through multidisciplinary consultation or collaboration with higher‐level hospitals. Additionally, eligible patients should be encouraged to participate in clinical trials.
15		Before initiating the first systemic treatment for advanced (stage IV) breast cancer, county‐level hospitals should conduct comprehensive imaging evaluations using the same imaging modality at baseline. For new metastatic lesions, a repeat biopsy is recommended to clarify pathological characteristics.
16		For patients in the terminal stage of breast cancer receiving palliative care, county‐level hospitals should conduct a comprehensive assessment of the patient's general condition, identify the cause of disease, and provide appropriate supportive treatment. Hospitals facing challenges in diagnosis and treatment should seek consultation with hospitals at the same or higher level within the county.
17	Recurrence monitoring	Re‐examinations for early‐stage breast cancer can be conducted at county‐level hospitals but are recommended at the hospital where the patient received anticancer treatment. Patients with stable disease should also be enrolled in chronic disease management programs at community or township health centers to receive routine monitoring, rehabilitation guidance, and reminders for periodic re‐examinations.
18	Follow‐up	Since patients in rural areas may face challenges in transportation, it is recommended to supplement in‐person follow‐ups with telephone or online consultations to closely monitor changes in their health status.

## 5 Challenges and Prospects

The current deepening of the tiered diagnosis and treatment system for breast cancer faces systemic challenges: Patients and primary healthcare institutions lack understanding of the concept of tiered diagnosis and treatment, and the principle of “primary care first” has not yet been universalized. There are significant shortcomings in information technology development, with cross‐level data sharing mechanisms lacking. A lack of pathology and imaging resources at the primary level leads to inefficient remote collaboration. There is a significant shortage of primary healthcare professionals, with pathology and radiotherapy specialists in county‐level medical institutions weak, and only a few capable of independently performing breast‐conserving surgery. Imbalances in resource coordination exacerbate barriers to continuity of care, limited access to targeted drugs at the primary level, and disparities in medical insurance reimbursement hindering the implementation of two‐way referrals. To address this, we can learn from the UK's general practitioner “gatekeeper system” to strengthen primary care authority, reference the US MD Anderson Center's information platform to achieve seamless transitions between community and regional centers, and emulate Japan's legislation to clarify institutional responsibilities and share medical insurance reimbursement.

The future requires multi‐faceted breakthroughs: Integrate the tiered approach through community health education and physician training, establishing referral incentives; accelerate the integration of provincial oncology information platforms, develop AI‐powered decision‐making modules to promote mutual recognition of examinations; implement a “county‐managed, township‐employed” staffing reform to cultivate specialized talent and explore the multi‐location practice of tertiary hospital experts; and simultaneously implement comprehensive, packaged payment for single‐disease medical insurance and the development of regional drug dispensing centers. Ultimately, we will achieve a leap forward in both quality of life and efficiency of medical resources as envisioned by “Healthy China 2030.”

## Author Contributions

**Jiani Wang:** writing – original draft, writing – review and editing. **Shuping Wang:** writing – original draft. **Erdan Huang:** writing – review and editing. **Fei Ma:** writing – review and editing.

## Ethics Statement

The authors have nothing to report.

## Consent

The authors have nothing to report.

## Conflicts of Interest

Professor Fei Ma is the member of the *Cancer Innovation* Editorial Board. To minimize bias, he was excluded from all editorial decision‐making related to the acceptance of this article for publication. The remaining authors declare no conflicts of interest.

## Supporting information


**Appendix 1.** Evaluation metrics for tiered diagnosis and treatment in breast cancer demonstration centers. **Appendix 2.** Evaluation metrics for tiered diagnosis and treatment in breast cancer standard centers. **Appendix 3.** Evaluation metrics for tiered diagnosis and treatment in cancer prevention and treatment centers.

## Data Availability

Data sharing is not applicable to this article as no data sets were generated or analyzed during the current study.
