# Supplementary material for: Expert Consensus on Tiered Diagnosis and Treatment of Breast Cancer as a Single‐Disease Management Model in China (2025 Edition)
Source: Cancer Innov. 2025 Oct 23;4(5):e70031. doi: 10.1002/cai2.70031 (PMC12547835; doi:10.1002/cai2.70031)
Supplement: Supplementary file 1 — Appendix 1. Evaluation metrics for tiered diagnosis and treatment in breast cancer demonstration centers. Appendix 2. Evaluation metrics for tiered diagnosis and treatment in breast cancer standard centers. Appendix 3. Evaluation metrics for tiered diagnosis and treatment in cancer prevention and treatment centers. [file CAI2-4-e70031-s001.docx]

### Appendix 1. Evaluation metrics for tiered diagnosis and treatment in breast cancer demonstration centers

#### 1. Management Metrics

1.1 Rate of complications in patients undergoing breast cancer surgery

1) Definition: The proportion of breast cancer patients experiencing postoperative complications among all discharged breast cancer patients undergoing surgery within the same period.

2) Formula:$Rate of complications in patients undergoing breast cancer surgery=\frac{\sum Number of patients with complications following breast cancer surgery}{\sum Number of patients discharged following breast cancer surgery within the same period}\times100\%$

3) Significance: To measure medical technical and management capabilities associated with breast cancer surgery.

4) Note: Patients undergoing palliative resection are excluded.

1.2 Rate of Class I surgical site infections in breast cancer surgeries

1) Definition: The proportion of breast cancer patients with Class I surgical site infections among all breast cancer patients with Class I surgical incisions within the same period.

2) Formula:$Rate of Class I surgical site infections in breast cancer surgeries=\frac{\sum Number of patients with Class I surgical site infection following breast cancer surgery}{\sum Number of patients with Class I surgical incisions following breast cancer surgery within the same period}\times100\%$

3) Significance: To reflect the hospital's management, prevention, and control of infections in patients with Class I incisions.

4) Note: Patients with “Grade C” grade of healing on the first page of the medical record are included in the “Number of patients with Class I surgical site infections”.

1.3 Rate of unplanned reoperation in breast cancer patients

1) Definition: The proportion of breast cancer patients undergoing unplanned reoperation among all breast cancer patients undergoing surgery within the same period.

2) Formula:$Rate of unplanned reoperation in breast cancer patients=\frac{\sum Number of breast cancer patients undergoing unplanned reoperation}{\sum Number of patients undergoing breast cancer surgery within the same period}\times100\%$

3) Significance: To reflect the medical technical capability and quality of care.

4) Note: Unplanned reoperation refers to a second surgical procedure performed at the same site due to bleeding, incision infection, or incision dehiscence during the current hospitalization.

1.4 Mortality rate among low-risk breast cancer patients

1) Definition: Patients at low risk are identified using Diagnosis-Related Groups (DRGs), and the mortality rate is calculated as the proportion of deaths within this group.

2) Formula:$Mortality rate among low-risk breast cancer patients=\frac{\sum Number of deaths in low-risk breast cancer patients}{\sum Number of low-risk breast cancer patients}\times100\%$

3) Significance: To measure the safety and quality of services provided to inpatients.

4) Note: Low-risk breast cancer patients: Patients with a mortality rate lower than one standard deviation below the mean are classified into the low-risk DRG. In this group, deaths are more likely related to the clinical management process rather than the disease itself.

#### 2. Diagnostic metrics

2.1 Rate of clinical TNM staging in patients with breast cancer prior to first treatment:

1) Definition: The proportion of breast cancer patients who completed clinical TNM staging prior to first treatment among all breast cancer patients receiving first treatment within the same period.

2) Formula: Rate of clinical TNM staging in patients with breast cancer prior to first treatment$=\frac{\sum Number of breast cancer patients who completed clinical TNM staging prior to first treatment}{\sum Number of breast cancer patients receiving first treatment within the same period}\times100\%$

3) Significance: To comprehensively assess the patient's condition before treatment and to standardize anti-cancer treatment.

4) Note: Clinical TNM staging should generally be completed within 30 days prior to the first treatment.

2.2 Rate of standardization in clinical TNM staging in patients with breast cancer prior to first treatment

1) Definition: The proportion of breast cancer patients who completed standardized clinical TNM staging prior to first treatment among all breast cancer patients receiving first treatment within the same period.

2) Formula:$Rate of standardization in clinical TNM staging in patients with breast cancer prior to first treatment=\frac{\sum Number of breast cancer patients who completed who copmleted standardized clinical TNM staging prior to first treatment}{\sum Number of breast cancer patients receiving first treatment within the same period}\times100\%$

3) Significance: To comprehensively assess the patient's condition before treatment and to standardize anti-cancer treatment.

4) Note: Standardization in clinical TNM staging refers to the use of either Basic Strategy 1 or Basic Strategy 2. Basic Strategy 1: Breast ultrasound or mammography or breast MRI + chest CT + abdominal ultrasound (or abdominal CT or abdominal MRI); Basic Strategy 2: Breast ultrasound or mammography or breast MRI + PET-CT. Clinical TNM staging should generally be completed within 30 days prior to the first treatment.

2.3 Rate of pathological diagnosis in patients with breast cancer prior to anti-cancer drug therapy

1) Definition: The proportion of breast cancer patients with a pathological diagnosis prior to anti-cancer drug therapy among all breast cancer patients receiving first anti-cancer drug therapy within the same period.

2) Formula:$Rate of pathological diagnosis in patients with breast cancer prior to anti-cancer drug therapy=\frac{\sum Number of breast cancer patients who received pathological diagnosis prior to anti-cancer drug therapy}{\sum Number of breast cancer patients receiving first anti-cancer drug therapy within the same period}\times100\%$

3) Significance: To determine the pathological diagnosis and to support the formulation of a multimodal treatment plan.

4) Note: Anti-cancer drug therapy includes chemotherapy, targeted therapy, immunotherapy, and endocrine therapy.

2.4 Rate of postoperative pathology report completeness in patients with breast cancer

1) Definition: The proportion of breast cancer patients with complete postoperative pathology reports among all breast cancer patients undergoing surgery within the same period.

2) Formula:$Rate of postoperative pathology report completeness in patients with breast cancer=\frac{\sum Number of breast cancer patients with complete postoperative pathology reports}{\sum Number of breast cancer patients with postoperative pathology reports within the same period}\times100\%$

3) Significance: To determine the standardization of pathology reports and to guide postoperative adjuvant therapy for breast cancer.

2.5 Rate of pathological diagnosis in patients with breast cancer prior to radiotherapy

1) Definition: The proportion of breast cancer patients with a confirmed pathological diagnosis prior to radiotherapy among all breast cancer patients receiving radiotherapy within the same period.

2) Formula:$Rate of pathological diagnosis in patients with breast cancer prior to radiotherapy=\frac{\sum Number of breast cancer patients with confirmed pathological diagnosis prior to radiotherapy}{\sum Number of breast cancer patients receiving radiotherapy within the same period}\times100\%$

3) Significance: To evaluate the standardization of treatment for breast cancer patients receiving radiotherapy.

2.6 Rate of ultrasound correlation in patients with breast cancer prior to first treatment

1) Definition: The proportion of breast cancer patients with an ultrasound diagnosis of BI-RADS category 4 or higher prior to first treatment among all patients with pathologically confirmed breast cancer.

2) Formula:$Rate of ultrasound correlation in patients with breast cancer prior to first treatment=\frac{\sum Number of breast cancer patients with an ultrasound diagnosis of BI-RADS category 4 or higher prior to first treatment}{\sum Number of breast cancer patients with pathological diagnosis}\times100\%$

3) Significance: To demonstrate the consistency between ultrasound and pathological diagnoses and the standardization of ultrasound diagnosis of breast cancer. This is an important reference metric for evaluating the patient's condition before treatment.

4) Note: Breast ultrasound should generally be completed within 30 days before treatment.

2.7 Rate of mammographic correlation in patients with breast cancer prior to first treatment

1) Definition: The proportion of breast cancer patients with a mammographic diagnosis of BI-RADS category 4 or higher prior to first treatment among all breast cancer patients with pathological diagnosis.

2) Formula:$Rate of mammographic correlation in patients with breast cancer prior to first treatment=\frac{\sum Number of breast cancer patients with a mammographic diagnosis of BI-RADS category 4 or higher prior to first treatment}{\sum Number of breast cancer patients with pathological diagnosis}\times100\%$

3) Significance: To reflect the consistency between mammographic and pathological diagnoses and the standardization of mammographic diagnosis of breast cancer. This is an important reference metric for evaluating the patient's condition before treatment.

4) Note: Mammography should generally be completed within 30 days before treatment.

#### 3. Surgical Metrics

3.1 Proportion of patients with early-stage breast cancer undergoing sentinel lymph node biopsy

1) Definition: The proportion of patients with early-stage breast cancer (clinical stage: T1-2N0M0) undergoing SLNB among all patients with early-stage breast cancer undergoing surgery within the same period.

2) Formula:$Proportion of patients with early-stage breast cancer undergoing sentinel lymph node biopsy=\frac{\sum Number of patients with early-stage breast cancer undergoing sentinel lymph node biopsy}{\sum Number of patients with early-stage breast cancer undergoing surgery within the same period}\times100\%$

3) Significance: To reflect the application of SLNB in patients with early-stage, axillary lymph node-negative disease (clinical stage: T1-2N0M0).

4) Note: Breast cancer surgery and SLNB are not necessarily performed within the same period.

3.2 Proportion of patients with ≥ 10 axillary lymph nodes dissected

1) Definition: The proportion of breast cancer patients with ≥ 10 axillary lymph nodes dissected during surgery among all breast cancer patients undergoing axillary lymph node dissection during surgery.

2) Formula:$Proportion of patients with \geq10 axillary lymph nodes dissected=\frac{\sum Number of breast cancer patients with \geq10 axillary lymph nodes dissected during surgery}{\sum Number of breast cancer patients undergoing axillary lymph node dissection during surgery}\times100\%$

3) Significance: To assess the standardization of intraoperative axillary lymph node dissection for breast cancer patients.

4) Note: For breast cancer patients indicated for axillary lymph node dissection, at least 10 axillary lymph nodes should be dissected during surgery to truthfully reflect the condition of the axillary lymph nodes.

#### 4. Radiotherapy

4.1 Proportion of patients with breast cancer receiving postoperative radiotherapy after breast-conserving surgery

1) Definition: The proportion of breast cancer patients undergoing radiotherapy after breast-conserving surgery among all breast cancer patients who should receive radiotherapy after breast-conserving surgery.

2) Formula:$Proportion of patients with breast cancer receiving postoperative radiotherapy after breast-conserving surgery=\frac{\sum Number of breast cancer patients receiving radiotherapy after breast-conserving surgery}{\sum Number of breast cancer patients who should receive radiotherapy after breast-conserving surgery}\times100\%$

3) Significance: To determine the standardization of radiotherapy after breast-conserving surgery for breast cancer patients.

4) Note: If the patient does not receive adjuvant chemotherapy, radiotherapy should generally be initiated within 60 days after surgery. If the patient receives adjuvant chemotherapy, radiotherapy should generally be initiated within 60 days after the completion of the last chemotherapy cycle. Regardless of whether adjuvant chemotherapy is administered, radiotherapy should generally be completed within six months after surgery.

4.2 Proportion of patients with breast cancer receiving postoperative radiotherapy after modified radical mastectomy

1) Definition: The proportion of patients with T3-4 tumors or ≥ 4 metastatic lymph nodes who received radiotherapy after modified radical mastectomy among all patients with T3-4 tumors or ≥ 4 metastatic lymph nodes after modified radical mastectomy who should receive radiotherapy.

2) Formula:$Proportion of patients with breast cancer receiving postoperative radiotherapy after modified radical mastectomy=\frac{\sum Number of breast cancer patients with T3-4 tumors or \geq4 metastatic lymph nodes who received radiotherapy after modified radical mastectomy}{\sum Number of breast cancer patients with T3-4 tumors or \geq4 metastatic lymph nodes who should receive radiotherapy after modified radical mastectomy}\times100\%$

3) Significance: To evaluate the standardization of treatment for breast cancer patients after modified radical mastectomy.

4.3 Compliance rate of radiotherapy records for breast cancer patients

1) Definition: The proportion of breast cancer patients with recorded radiation approach, target volume definition, and radiation dose among all breast cancer patients undergoing radiotherapy within the same period.

2) Formula:$Compliance rate of radiotherapy records for breast cancer patients=\frac{\sum Number of breast cancer patients with recorded radiotherapy approach,target volume, and radiation dose}{\sum Number of breast cancer patients undergoing radiotherapy within the same period}\times100\%$

3) Significance: Radiotherapy approach, target volume definition, and radiation dose are key radiotherapy metrics for assessing the standardization of treatment for breast cancer. They are also important reference metrics for evaluating the possibility of repeat radiotherapy and the occurrence of radiotherapy complications.

#### 5. Pharmacological Therapy

5.1 Proportion of patients with clinical stage III breast cancer receiving neoadjuvant therapy prior to surgery

1) Definition: The proportion of patients with clinical stage III (excluding T3N1M0) breast cancer receiving neoadjuvant therapy prior to surgery among all inpatients with clinical stage III (excluding T3N1M0) breast cancer undergoing surgery within the same period.

2) Formula:$Proportion of patients with clinical stage III breast cancer receiving neoadjuvant therapy prior to surgery=\frac{\sum Number of patients with clinical stage III (excluding T3N1M0) breast cancer who receive neoadjuvant therapy before surgery}{\sum Number of patients with clinical stage III (excluding T3N1M0) breast cancer undergoing surgery within the same period}\times100\%$

3) Significance: To determine the standardization of treatment for patients with locally advanced breast cancer.

4) Note: If the patient receives neoadjuvant therapy, the last treatment should be completed within 30 days prior to surgery.

5.2 Compliance rate of chemotherapy records for breast cancer patients

1) Definition: The proportion of breast cancer patients with recorded chemotherapy regimen, chemotherapy dosage, and chemotherapy duration among all breast cancer patients receiving chemotherapy within the same period.

2) Formula:$Compliance rate of chemotherapy records for breast cancer patients=\frac{\sum Number of breast cancer patients with recorded chemotherapy regimen, dosage, and duration}{\sum Number of breast cancer patients undergoing chemotherapy within the same period}\times100\%$

3) Significance: Chemotherapy regimen and dosage are key metrics of chemotherapy and are important reference metrics for evaluating the standardization of treatment and formulating subsequent treatment plans.

4) Note: Adjuvant chemotherapy should generally be initiated within 60 days after surgery.

5.3 Proportion of patients with advanced metastatic (clinical stage M1) breast cancer receiving systemic treatment as initial treatment

1) Definition: The proportion of patients with advanced metastatic breast cancer receiving systemic therapy as first treatment among all patients with advanced metastatic breast cancer receiving first treatment within the same period.

2) Formula:$Proportion of patients with advanced metastatic (clinical stage M1) breast cancer receiving systemic treatment as initial treatment=\frac{\sum Number of patients with advanced metastatic breast cancer receiving systemic therapy as first treatment}{\sum Number of patients with advanced metastatic breast cancer receiving first treatment within the same period}\times100\%$

3) Significance: To reflect the standardization of treatment for advanced breast cancer.

5.4 Proportion of postoperative hormone receptor-positive patients with breast cancer receiving adjuvant endocrine therapy

1) Definition: The proportion of postoperative hormone receptor-positive patients with breast cancer receiving endocrine therapy among all postoperative hormone receptor-positive patients with breast cancer who should receive endocrine therapy within the same period.

2) Formula:$Proportion of postoperative hormone receptor-positive patients with breast cancer receiving adjuvant endocrine therapy=\frac{\sum Number of patients with postoperative hormone receptor-positive breast cancer receiving endocrine therapy}{\sum Number of patients with postoperative hormone receptor-positive breast cancer within the same period}\times100\%$

3) Significance: To reflect the standardization of postoperative treatment for breast cancer.

4) Note: ER expression ≥ 1% (ER-positive) and/or PR expression ≥ 1% (PR-positive). If the patient does not receive adjuvant chemotherapy, endocrine therapy should generally be initiated within 30 days and no later than 60 days after surgery. If the patient receives adjuvant chemotherapy, endocrine therapy should be initiated within 30 days and no more than 60 days after the completion of chemotherapy.

5.5 Proportion of HER2-positive patients with breast cancer receiving postoperative targeted therapy

1) Definition: The proportion of patients with postoperative HER2-positive breast cancer (primary lesion diameter ≥ 1 cm or positive lymph nodes) receiving targeted therapy with trastuzumab among all patients with postoperative HER2-positive breast cancer (primary lesion diameter ≥ 1 cm or positive lymph nodes) who should receive targeted therapy.

2) Formula:$Proportion of HER2-positive patients with breast cancer receiving postoperative targeted therapy=\frac{\sum Number of patients with postoperative HER2-positive breast cancer (primary lesion diameter \geq1 cm or lymph node-positive) receiving targeted therapy within six months}{\sum Number of patients with postoperative HER2-positive breast cancer (primary lesion diameter \geq1 cm or positive lymph nodes) who should receive targeted therapy}\times100\%$

3) Significance: To reflect the standardization of postoperative treatment for breast cancer.

4) Note: HER2 positivity is defined as immunohistochemistry (IHC) 3+ or IHC 2+ and fluorescence in situ hybridization (FISH)-positive.

5.6 Proportion of hormone receptor-positive patients with breast cancer receiving postoperative adjuvant endocrine therapy

1) Definition: The proportion of postoperative hormone receptor-positive patients with breast cancer receiving endocrine therapy among all breast cancer patients receiving postoperative adjuvant endocrine therapy within the same period.

2) Formula:$Proportion of hormone receptor-positive patients with breast cancer receiving postoperative adjuvant endocrine therapy=\frac{\sum Number of patients with postoperative hormone receptor-positive breast cancer receiving endocrine therapy}{\sum Number of breast cancer patients receiving postoperative adjuvant endocrine therapy within the same period}\times100\%$

3) Significance: To reflect the standardization of postoperative treatment for breast cancer.

4) Note: Hormone receptor positivity is defined as ER and/or PR positivity with a percentage of IHC-positive nuclei of at least 1%.

5.7 Proportion of HER-2 positive patients with breast cancer receiving anti-HER2 targeted therapy

1) Definition: The proportion of HER2-positive patients with breast cancer receiving anti-HER2 targeted therapy among all breast cancer patients receiving anti-HER2 targeted therapy within the same period.

2) Formula:$Proportion of HER2-positive patients among all breast cancer patients receiving anti-HER2 targeted therapy=\frac{\sum Number of HER2-positive patients receiving anti-HER2 targeted therapy}{\sum Number of breast cancer patients receiving anti-HER2 targeted therapy within the same period}\times100\%$

3) Significance: To evaluate the standard use of anti-HER2 targeted therapy.

4) Note: HER2 positivity is defined as IHC 3+ or IHC 2+ and FISH-positive.

#### 7. Other Metrics

7.1 Proportion of breast cancer patients undergoing MDT evaluation prior to first treatment

1) Definition: The proportion of breast cancer patients undergoing MDT evaluation prior to first treatment among all breast cancer patients receiving first treatment within the same period.

2) Formula:$Proportion of patients with breast cancer undergoing MDT evaluation prior to first treatment=\frac{\sum Number of breast cancer patients undergoing MDT evaluation prior to first treatment}{\sum Number of breast cancer patients receiving first treatment within the same period}\times100\%$

3) Significance: To serve as an important process metric to ensure standardized and individualized anti-cancer treatment.

4) Note: This metric is not assessed at this time.

7.2 Rate of post-treatment follow-up of breast cancer patients

1) Definition: The proportion of inpatients with breast cancer who completed follow-up visits within N years post-treatment among all inpatients with breast cancer receiving treatment within the same period.

2) Formula:$Post-treatment follow-up rate of breast cancer patients=\frac{\sum Number of breast cancer patients who completed follow-up visits within N years post-treatment}{\sum Number of breast cancer patients within the same period}\times100\%$

3) Significance: To reflect the hospital's long-term management of breast cancer patients and to provide a basis for further evaluating the quality control metrics.

4) Note: This metric is not assessed at this time.

### Appendix 2. Evaluation metrics for tiered diagnosis and treatment in breast cancer standard centers

#### 1. Management Metrics

1.1 Rate of Class I surgical site infections in breast cancer surgeries

1) Definition: The proportion of breast cancer patients with Class I surgical site infections among all breast cancer patients with Class I surgical sites.

2) Formula:$Rate of Class I surgical site infections in breast cancer surgeries=\frac{Number of breast cancer patients with Class I surgical site infection}{Total number of breast cancer patients with Class I surgical sites within the same period}\times100\%$

3) Significance: To reflect the hospital's capability in the management, prevention, and control of infections.

4) Note: Patients with “Grade C” healing on the first page of the medical record are included in the “Number of patients with Class I surgical incision infections”.

#### 2. Diagnostic metrics

2.1 Rate of clinical diagnosis by TNM staging in patients with breast cancer prior to first treatment

1) Definition: The proportion of breast cancer patients undergoing clinical diagnosis by TNM staging prior to first treatment among all breast cancer patients receiving first treatment.

2) Formula:$Rate of clinical diagnosis by TNM staging in patients with breast cancer prior to first treatment=\frac{Number of breast cancer patients undergoing clinical diagnosis by TNM staging prior to first treatment}{Total number of breast cancer patients receiving first treatment within the same period}\times100\%$

3) Significance: To comprehensively assess the patient's condition before treatment, supporting standardized anti-cancer treatment.

4) Note: Clinical diagnosis by TNM staging should generally be completed within 30 days prior to the first treatment.

2.2 Rate of standardization in clinical assessment of TNM stage in patients with breast cancer prior to first treatment

1) Definition: The proportion of breast cancer patients with standardized clinical assessment of TNM stage prior to the first treatment among all breast cancer patients receiving first treatment.

2) Formula:$Rate of compliance in clinical assessment by TNM staging in patients with breast cancer prior to first treatment=\frac{Number of breast cancer patients whose clinical assessment by TNM staging complied with one of the two basic strategies prior to first treatment}{Total number of breast cancer patients receiving first treatment within the same period}\times100\%$

3) Significance: To assess the standardization of disease assessment.

4) Note: Standardization in clinical assessment of TNM stage refers to with the use of either Basic Strategy 1 or Basic Strategy 2. Basic Strategy 1: Breast ultrasound/mammography or breast MRI + chest CT + abdominal ultrasound (or CT or MRI); Basic Strategy 2: Breast ultrasound/mammography or breast MRI + PET-CT.

2.3 Rate of pathological diagnosis in patients with breast cancer prior to non-surgical treatment

1) Definition: The proportion of breast cancer patients who received pathological diagnosis prior to first non-surgical treatment among all breast cancer patients receiving first non-surgical treatment.

2) Formula:$Rate of pathological diagnosis in patients with breast cancer prior to non-surgical treatment=\frac{Number of breast cancer patients with pathological diagnosis prior to first non-surgical treatment}{Total number of breast cancer patients receiving first non-surgical treatment within the same period}\times100\%$

3) Significance: To determine the pathological diagnosis and to support the selection of a multimodal anti-cancer treatment plan.

4) Note: Non-surgical treatment includes radiotherapy, chemotherapy, targeted therapy, endocrine therapy, and immunotherapy.

#### 3. Surgical Metrics

3.1 Rate of sentinel lymph node biopsy in patients with early-stage breast cancer

1) Definition: The proportion of patients with early-stage breast cancer undergoing sentinel lymph node biopsy among all patients with early-stage breast cancer undergoing surgery.

2) Formula:$Rate of sentinel lymph node biopsy in patients with early-stage breast cancer=\frac{Number of patients with early-stage breast cancer undergoing sentinel lymph node biopsy}{Total number of patients with early-stage breast cancer undergoing surgery within the same period}\times100\%$

3) Significance: To rationalize axillary lymph node dissection.

4) Note: Early-stage breast cancer refers to T1-2N0M0 breast cancer.

3.2 Rate of adequate axillary lymph node dissection

1) Definition: The proportion of breast cancer patients with ≥ 10 axillary lymph nodes dissected among all breast cancer patients undergoing axillary lymph node dissection.

2) Formula:$Rate of adequate axillary lymph node dissection=\frac{Number of breast cancer patients with \geq10 axillary lymph nodes dissected}{Total number of breast cancer patients undergoing axillary lymph node dissection within the same period}\times100\%$

3) Significance: To assess the standardization of intraoperative axillary lymph node dissection for breast cancer patients.

#### 4. Radiotherapy

4.1 Rate of radiotherapy after breast-conserving surgery in patients with breast cancer

1) Definition: The proportion of breast cancer patients undergoing radiotherapy after breast-conserving surgery among all breast cancer patients undergoing breast-conserving surgery.

2) Formula:$Rate of radiotherapy after breast-conserving surgery in patients with breast cancer=\frac{Number of breast cancer patients undergoing radiotherapy after breast-conserving surgery}{Total number of breast cancer patients undergoing breast-conserving surgery within the same period}\times100\%$

3) Significance: To reflect the standardization of treatment for breast cancer patients after breast-conserving surgery.

4.2 Rate of radiotherapy after modified radical mastectomy in patients with breast cancer

1) Definition: The proportion of breast cancer patients undergoing radiotherapy after modified radical mastectomy among all breast cancer patients undergoing modified radical mastectomy.

2) Formula:$Rate of radiotherapy after modified radical mastectomy in patients with breast cancer=\frac{Number of breast cancer patients undergoing radiotherapy after modified radical mastectomy}{Total number of breast cancer patients undergoing modified radical mastectomy within the same period}\times100\%$

3) Significance: To evaluate the standardization of treatment for breast cancer patients after modified radical mastectomy.

4) Note: This metric applies to patients with T3-4 tumors or ≥ 4 metastatic lymph nodes after modified radical mastectomy.

#### 5. Pharmacological Therapy

5.1 Rate of neoadjuvant therapy in patients with locally advanced breast cancer

1) Definition: The proportion of patients with locally advanced breast cancer receiving neoadjuvant therapy among all patients with locally advanced breast cancer.

2) Formula:$Rate of neoadjuvant therapy in patients with locally advanced breast cancer=\frac{Number of patients with locally advanced breast cancer receiving neoadjuvant therapy}{Total number of patients with locally advanced breast cancer within the same period}\times100\%$

3) Significance: To reflect the standardization of treatment for patients with locally advanced breast cancer.

4) Note: Locally advanced breast cancer refers to stage III (excluding T3N1M0) breast cancer. Neoadjuvant therapy includes chemotherapy, targeted therapy, endocrine therapy, and other therapeutic approaches.

5.2 Rate of systemic treatment as first treatment in patients with advanced metastatic breast cancer

1) Definition: The proportion of patients with advanced metastatic breast cancer receiving systemic therapy as first treatment among all patients with advanced metastatic breast cancer receiving first treatment.

2) Formula:$Rate of systemic treatment as initial treatment in patients with advanced metastatic breast cancer=\frac{Number of patients with advanced metastatic breast cancer receiving systemic therapy as first treatment}{Total number of patients with advanced metastatic breast cancer receiving first treatment within the same period}\times100\%$

3) Significance: To reflect the standardization of treatment for patients with advanced breast cancer.

4) Note: Advanced metastatic breast cancer refers to M1 breast cancer. Systemic treatment refers to anti-cancer drug therapy, including chemotherapy, targeted therapy, immunotherapy, and endocrine therapy.

5.3 Rate of postoperative adjuvant endocrine therapy in patients with hormone receptor-positive breast cancer

1) Definition: The proportion of patients with hormone receptor-positive breast cancer receiving postoperative adjuvant endocrine therapy among all patients with postoperative hormone receptor-positive breast cancer.

2) Formula:$Rate of postoperative adjuvant endocrine therapy in patients with hormone receptor-positive breast cancer=\frac{Number of patients with hormone receptor-positive breast cancer receiving postoperative adjuvant endocrine therapy}{Total number of patients with postoperative hormone receptor-positive breast cancer within the same period}\times100\%$

3) Significance: To reflect the standardization of treatment for patients with advanced breast cancer.

5.4 HER2-positive rate in patients with breast cancer receiving anti-HER2 targeted therapy

1) Definition: The proportion of HER2-positive breast cancer patients receiving anti-HER2 targeted therapy among all breast cancer patients receiving anti-HER2 targeted therapy.

2) Formula:$HER2-positive rate in patients with breast cancer receiving anti-HER2 targeted therapy=\frac{Number of HER2-positive breast cancer patients receiving anti-HER2 targeted therapy}{Total number of breast cancer patients receiving anti-HER2 targeted therapy within the same period}\times100\%$

3) Significance: To reflect the standard use of anti-HER2 targeted therapy.

### Appendix 3. Evaluation metrics for tiered diagnosis and treatment in cancer prevention and treatment centers

1. Rate of Clinical Stage Assessment Prior to First Treatment

1) Definition: The proportion of patients undergoing clinical stage assessment before first treatment among all patients receiving first treatment within the same period for ten cancer types (breast cancer, lung cancer, liver cancer, cervical cancer, gastric cancer, colorectal cancer, esophageal cancer, thyroid cancer, prostate cancer, and kidney cancer) that meet the inclusion criteria during the year.

2) Formula:$Rate of clinical staging prior to first treatment=\frac{Number of cancer patients undergoing clinical staging prior to first treatment}{Number of cancer patients receiving first treatment within the same period}\times100\%$

3) Significance: To comprehensively assess the patient's condition before treatment and to standardize anti-cancer treatment.

4) Note: Clinical diagnosis by TNM staging should generally be completed within 30 days prior to first treatment.

2. Rate of Postoperative TNM Staging

1) Definition: The proportion of patients undergoing postoperative pathological TNM staging among all patients undergoing surgery within the same period for eight cancer types (breast cancer, lung cancer, intrahepatic bile duct cancer, gastric cancer, colorectal cancer, thyroid cancer, prostate cancer, and kidney cancer) that meet the inclusion criteria during the year.

2) Formula:$Rate of postoperative pathological TNM staging=\frac{Number of cancer patients undergoing postoperative pathological TNM staging}{Number of cancer patients undergoing surgery within the same period}\times100\%$

3) Significance: To comprehensively assess the patient's condition before treatment and standardize anti-cancer treatment.

3. Rate of Pathological Diagnosis Prior to First Non-surgical Treatment

1) Definition: The proportion of patients undergoing pathological diagnosis prior to first non-surgical treatment among all patients receiving first non-surgical treatment within the same period for nine cancer types (breast cancer, lung cancer, intrahepatic bile duct cancer, cervical cancer, gastric cancer, colorectal cancer, esophageal cancer, prostate cancer, and kidney cancer) that meet the inclusion criteria during the year.

2) Formula:$Rate of pathological diagnosis prior to first non-surgical treatment=\frac{Number of cancer patients with pathological diagnosis prior to first non-surgical treatment}{Number of cancer patients receiving first non-surgical treatment within the same period}\times100\%$

3) Significance: To determine the pathological diagnosis and to support the formulation of a multimodal treatment plan.

4) Note: Non-surgical treatment includes radiotherapy, chemotherapy, targeted therapy, endocrine therapy, and immunotherapy.

4. Rate of Molecular Pathology Detection Prior to First Targeted Therapy/immunotherapy

1) Definition: The proportion of patients undergoing molecular pathology testing prior to first targeted therapy/immunotherapy among all patients receiving targeted therapy/immunotherapy within the same period for four cancer types (breast cancer, lung cancer, gastric cancer, and colorectal cancer) that meet the inclusion criteria during the year.

2) Formula:$Rate of molecular pathology detection prior to first targeted therapy/immunotherapy=\frac{Number of cancer patients who completed molecular pathology testing prior to first targeted therapy/immunotherapy}{Number of cancer patients receiving first targeted therapy/immunotherapy within the same period}\times100\%$

3) Significance: To strengthen medical quality management and to standardize clinical diagnosis and treatment, thereby promoting standardized and unified medical services.

5. Compliance Rate of Intraoperative Lymph Node Dissection

1) Definition: The proportion of patients with N lymph nodes dissected during surgery among all patients receiving lymph node dissection within the same period for five cancer types (breast cancer, lung cancer, liver cancer, colorectal cancer, and esophageal cancer) that meet the inclusion criteria during the year.

2) Formula:$Compliance rate of intraoperative lymph node dissection=\frac{Number of cancer patients who had lymph node dissection during surgery as required}{Number of patients with a certain cancer undergoing lymph node dissection during the same period}\times100\%$

3) Significance: To strengthen medical quality management and to standardize clinical diagnosis and treatment, thereby promoting standardized and unified medical services.
